# Long-term recovery of sensorimotor functions and prediction of participation in survivors of critical illness: a prospective cohort study

**DOI:** 10.1186/s40560-025-00808-9

**Published:** 2025-09-08

**Authors:** Johanna Weghorn, Melanie Finsterhölzl, Franziska Wippenbeck, Klaus Jahn, Marion Egger, Jeannine Bergmann

**Affiliations:** 1 Physiotherapy, School of Health Professions, Bern University of Applied Sciences, Bern, Switzerland; 2 German Center for Vertigo and Balance Disorders, Ludwig-Maximilians-Universitat (LMU), University Hospital Grosshadern, Munich, Germany; 3 Research Group, Department of Neurology, Schoen Clinic Bad Aibling, Kolbermoorer Strasse 72, 83043 Bad Aibling, Germany

**Keywords:** Critical illness, Post-intensive care syndrome, Intensive care unit-acquired weakness, Postural balance, Recovery of function, Social participation

## Abstract

**Background:**

Survivors of critical illness frequently face physical, cognitive and psychological impairments after intensive care. Sensorimotor impairments potentially have a negative impact on participation. However, comprehensive understanding of sensorimotor recovery and participation in survivors of critical illness is limited. Therefore, the aims of this study were to quantify long-term sensorimotor recovery in survivors of critical illness, to examine participation in daily life 1.5 years after illness onset, and to assess the predictive capacity of sensorimotor assessments for future participation.

**Methods:**

Survivors of critical illness who were mechanically ventilated ≥ 5 days on the ICU and who were admitted with weakness to neurorehabilitation were included in this single-center prospective cohort study. Time effects on sensation, muscle strength, balance, walking and dexterity were described at admission to and at discharge from rehabilitation, and 1.5 years after critical illness onset. Participation was assessed with the Reintegration to Normal Living Index. A multiple linear regression with sensorimotor outcomes at rehabilitation admission was conducted to find predictive associations with participation. The model was compared to an extended regression model containing demographic variables and factors known to be associated with participation or quality of life.

**Results:**

All sensorimotor outcomes among participants (n = 250, median age 63 (54–73) years) improved over time. However, in most patients some deficits remained after rehabilitation and on long-term follow-up. Good participation (≥ 75%) was achieved by 60.2% of survivors 1.5 years after critical illness onset. Concerning participation, the Mini Balance Evaluation Systems Test (Mini-BESTest) together with the Box-and-Block-Test, the Five-Times-Sit-to-Stand-Test, and the Medical Research Council score at rehabilitation admission formed a predictive model (R^2^ = 0.157, p < 0.001). The extended regression analysis resulted in a model (R^2^ = 0.357, p < 0.001) with the variables depression, duration of mechanical ventilation, cognitive function, Mini-BESTest, comorbidities, sex and cerebral lesion.

**Conclusions:**

We observed significant improvements in sensorimotor function, albeit with lingering deficits in sensation, strength, balance, dexterity and participation. Sensorimotor functions at rehabilitation start have limited explanatory power in predicting participation 1.5 years after disease onset.

*Trial registration* German Clinical Trial Register, DRKS00021753. Date of registration: September 03, 2020.

**Supplementary Information:**

The online version contains supplementary material available at 10.1186/s40560-025-00808-9.

## Background

Advancements in critical care have markedly improved survival rates among patients with life-threatening diseases [1, 2]. However, many survivors of critical illness face persistent physical, cognitive, and psychological impairments, collectively termed post-intensive care syndrome (PICS) [2, 3]. The physical component of PICS primarily manifests as intensive care unit (ICU)-acquired weakness (ICUAW), which affects up to 60% of survivors and is characterized by persistent weakness following ≥ 5 to 7 days of mechanical ventilation, assessed by the Medical Research Council (MRC) Sum Score (< 48/60) [2, 4–6]. Prolonged mechanical ventilation, extended ICU stays, immobilization, and conditions like sepsis or other inflammatory or infectious responses are key risk factors for developing ICUAW, leading to sensorimotor impairment, frailty, and increased mortality [1, 2, 4, 5, 7, 8]. ICUAW at ICU discharge is associated with prolonged inpatient care and poorer functional outcomes [4, 5, 8, 9]. Moreover, residual weakness at rehabilitation discharge correlates with reduced quality of life and lower 5-year survival rates [10].

Importantly, beyond basic physical recovery, participation—defined as engagement in life roles and meaningful activities including work, social life, and domestic responsibilities—is a crucial component of long-term recovery and associated with quality of life [11–13]. Participation is a primary goal in rehabilitation and is associated with reduced disability and mortality risk [11, 14, 15]. However, despite its relevance, little is known about participation outcomes among survivors of critical illness. Prior research has focused largely on cognitive [9] and sensory factors [16] influencing participation while studies in other neurological populations suggest that motor function, balance, and independence in mobility are also key determinants of participation [17–20].

Although muscle weakness is a well-established consequence of prolonged ICU stays, our understanding of the broader sensorimotor impairments, such as sensory deficits, balance disturbances, and mobility limitations, and their direct association with participation outcomes are under-researched. Most existing studies are retrospective, limited to short-term follow-up periods [5], or rely on telephone interviews and written, self-reported questionnaires rather than objective, performance-based assessments [9, 16, 21–23]. Moreover, existing prediction models for survivors of critical illness often prioritize endpoints like mortality or global disability, while participation and physical activity are underexplored [9, 23, 24]. A more comprehensive and longitudinal understanding of how sensorimotor functions recover over time—and how early sensorimotor performance predicts long-term participation—is essential for tailoring effective treatment strategies and establishing realistic goals for patients, families, and healthcare providers [5, 25].

Therefore, the aim of this cohort study was to investigate the long-term recovery trajectories of sensorimotor functions, including strength, balance, and sensation, and to determine their predictive value for participation 1.5 years after critical illness onset. We hypothesized that although sensorimotor function improves over time, deficits remain both at rehabilitation discharge and long-term follow-up. Furthermore, we posited that early rehabilitation assessments of physical function would predict participation outcomes at 1.5 years.

## Methods

### Study design

This study constitutes a subanalysis of data acquired from the prospective single-center cohort study titled “Critical Illness Polyneuropathy and Myopathy: Outcome, Predictors and Longitudinal Trajectories” (CINAMOPS) and was conducted at an inpatient neurorehabilitation center, the Schoen Clinic Bad Aibling, Germany. The study protocol was previously described [26]. The CINAMOPS study was approved by the Ludwig-Maximilian-University Ethic Commission in Munich according to the Declaration of Helsinki (project No. 20-166) and registered at the German Register for clinical studies as DRKS00021753.

**Fig. 1:**
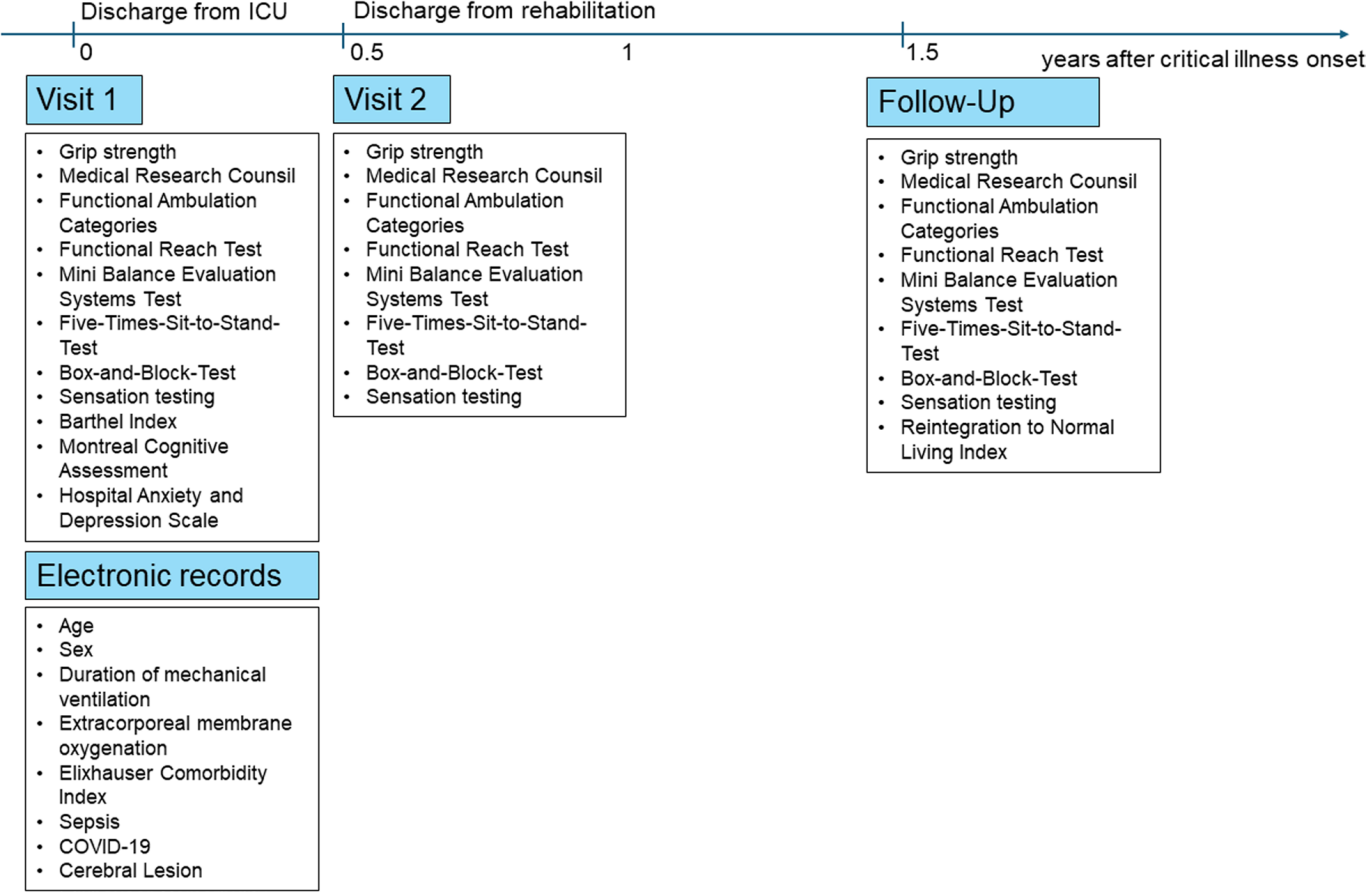
Study design of the subanalysis

For the purposes of the analysis presented here, data from three study visits were utilized: Visit 1 (V1) conducted within 2 weeks after ICU discharge, Visit 2 (V2) at discharge from inpatient rehabilitation, and a subsequent follow-up (FU) assessment at 1.5 years post-onset of critical illness. These visits were conducted in person by physical therapists and clinical researchers and included assessments of sensation, strength, balance, and walking ability. The assessors were extensively trained in using the different outcome measures to minimize the risk of bias. A graphical illustration of the data used in this analysis is presented in Fig. 1.

### Participants

All patients (≥ 18 years) who were mechanically ventilated for ≥ 5 days and fulfilled inclusion and exclusion criteria were asked to participate in the CINAMOPS study upon admission to rehabilitation. Exclusion criteria were full motor functioning (MRC sum score = 60/60), palliative treatment, or the presence of neuromuscular or neurological diseases causing muscular weakness itself. Additionally, participants needed to be awake and have sufficient (German) communication skills to complete the questionnaires. Written informed consent was obtained from all participants (or their legal guardians).

During their inpatient neurological rehabilitation, patients received a comprehensive rehabilitation program (standard care), which typically included approximately 100 min of functional therapies per day, including physiotherapy, respiratory and occupational therapy, dysphagia management, and neuropsychology. The type and amount of the various therapies were tailored to the patients' needs and deficits.

### Outcome measures

From electronic medical records the following patient demographics and details regarding ICU treatment were extracted: age at disease onset, length of ICU stay, duration of mechanical ventilation, extracorporeal membrane oxygenation (ECMO), primary disease, and the Elixhauser Comorbidity Index [27]. Additionally, information about the preclinical status, including the Functional Ambulation Categories [28], Clinical Frailty Scale [29], Barthel Index [30], known somatosensory impairment, living conditions, relationship status and employment status was gathered during the initial study visit.

Participation was assessed at the FU using the Reintegration to Normal Living Index (RNLI) [31]. This widely utilized and systematically developed tool comprises 11 statements regarding common social activities following neurological or traumatic incidents and was used in the German research version. This version has been previously employed in individuals with ICUAW and sepsis survivors [9, 13]. The items relate to indoor and community mobility, self-care, daily and recreational activities, the perception of self, roles and personal relationships [31, 32]. Previous studies have demonstrated good test–retest reliability, high internal consistency, and poor to good construct validity [32]. Responses in the research version are recorded on a three-point scale: agree, partially agree, do not agree. The maximum score of 22 points was converted to a 100% scale to facilitate comparison with other response formats [32]. A cut-off ≥ 75% was used for the definition of good participation as it was previously applied in individuals with ICUAW [9]. Missing values in this assessment were replaced by last observation carried forward.

At each of the three timepoints (V1, V2, FU), eight sensorimotor outcomes were evaluated. Grip strength of both arms was measured in two trials using a Jamar hand-held dynamometer (Kern MAP 130K1, Balingen, Germany). The maximum value obtained from these trials was compared to a reference value adjusted for the patient's age, sex, and height [33], yielding the proportion (in %) of the strongest grip strength. Grip strength is particularly recommended as an outcome measure in ICU and elderly populations and serves as an informative biomarker, given its association with functional limitations and its predictive link to all-cause and disease-specific mortality, future function, bone mineral density, cognition, and depression [33].

Muscle strength was assessed using the manual MRC score for shoulder abduction, elbow flexion, hand dorsal extension, hip flexion, knee extension, and ankle dorsal extension. These scores were summed to derive a total sum score, with separate average scores calculated for the upper and lower extremities to provide a comprehensive view of sensorimotor function recovery. Excellent interrater agreement on the MRC sum-score was observed, facilitating the quantification of global muscle strength between 0 and 60 in patients with ICUAW [34].

Sensory testing included superficial sensation, proprioception, and vibration sense. Superficial sensation was assessed with light touch on the left and right region of the biceps brachii, the palmar hand, the thigh, and the plantar foot, while proprioception was tested with the motion sense in the thumb and ankle [35]. No (0) /impaired (1) /intact sensation (2) generated maximum scores indicating ‘no superficial sensation impairment’ (16/16 points) and ‘no proprioceptive deficits’ (8/8 points) accordingly. Vibration sense was tested using a graduated tuning fork with a range from 0 to 8 in the upper limb at the ulnar wrist, in the lower limb at the big toe. Average scores of the obtained values were compared to normative data [36] and were defined as within normal range in the upper limb above 6 and in the lower limb above 4 points. Sensory deficits were categorized as binary outcomes (1 for deficits, 0 for absence of deficits) both collectively and for each phenotype and extremity separately.

Walking ability was classified using the Functional Ambulation Categories (FAC) [28], a 6-point scale grading the extent of assistance required for walking. A score of 0 indicates that a patient is a non-ambulator or needs assistance from more than two people to walk. Scores from 1 to 3 indicate varying levels of assistance or supervision from one person. A score of 4 and 5 is given to those who can walk without any assistance or supervision either indoors on even surfaces or outdoors including stairs and slopes.

Balance was assessed using the Functional Reach Test [37] and the Mini Balance Evaluation Systems Test (Mini-BESTest) [38]. The Functional Reach Test is a simple bedside test measuring dynamic balance performance by quantifying the distance between the length of an outstretched arm in a maximal forward reach, while maintaining a fixed base of support. This outcome provides insights into balance impairment and fall risk [37]. The Mini-BESTest, a 14-item test scored on a 3-level ordinal scale (0–2) evaluates various aspects of balance including (1) anticipatory postural adjustments; (2) reactive postural control; (3) sensory orientation and (4) dynamic gait. Recent studies have demonstrated excellent reliability and validity of the Mini-BESTest in survivors of critical illness [39].

The Five-Times-Sit-to-Stand-Test (5xSST) assesses lower limb strength and balance by measuring the time taken to complete five repetitions of standing up and sitting down [40]. Participants unable to complete the test were excluded from median and mixed model calculations.

The Box-and-Block-Test [41] is a frequently used baseline measurement for gross manual dexterity in neurorehabilitation and upper limb impairment. It has an excellent test–retest and interrater reliability, as well as content validity [41]. Its maximal obtained value was compared to the sex- and age-matched reference values of a healthy population [42].

### Statistical analysis

Continuous data are presented as means (± SD) or medians (quartile 1 to quartile 3), while categorical variables are expressed as n (%), as appropriate. Normal distribution was assessed visually and by Shapiro–Wilk test.

For sensorimotor outcomes, the estimation of time effects across the three time points was done using a linear mixed model for continuous outcomes and a generalized mixed-effects model for the dichotomous sensory outcomes. Time was modeled as a fixed categorical effect, as models with time as a categorical variable provided a better fit than those using time as a continuous variable (linear or non-linear). Subjects were included as random effects, with both random intercepts and random slopes for time specified in the linear models to account for individual differences in change over time. In the generalized mixed models, only random intercepts per subject were included to ensure model stability. Missing data were assumed to be missing at random [43]. A global test of the time effect was conducted using Type III analysis of variance (ANOVA) with Satterthwaite´s approximation for degrees of freedom. Pairwise post hoc comparisons were performed using Tukey-adjusted p-values to control for multiple comparisons.

Correlations between sensorimotor outcomes at FU and the RNLI at FU were calculated prior to investigate the association between sensorimotor outcomes at V1 and the RNLI. To determine the association between physical assessment and participation 1.5 years after disease onset, a multiple linear regression analysis was conducted utilizing the variable selection algorithm recommended by Heinze et al. [44]. The algorithm starts with a full model including all predictors, followed by stepwise backward elimination using the Akaike information criterion (AIC) to determine the optimal set of variables. Stability and variable sensitivity of the selected model were assessed by bootstrapping, in which the dataset was resampled with replacement and the model re-fitted 1000 times. This procedure provides inclusion frequencies for each predictor variable, sampling distributions of regression coefficients, and other measures of model stability. Only datasets with complete visit 1 and RNLI score at FU were included in the regression analysis.

In a first step, the variable selection algorithm was applied to reduce the full model, which included all sensorimotor outcome measures at V1, namely: MRC sum score, 5xSST, sensation (summarizing deficits of light touch, proprioception and vibration tests), maximal Box-and-Block-Test (in % of reference), maximal grip strength (in % of reference), Functional Reach Test, Mini-BESTest total score and FAC. For the multiple linear regression model, the categorical FAC was transformed to a dichotomous outcome by summarizing the scores 0–3 and 4–5 to groups of non-independent and independent walkers [45]. The time values of the 5xSST were converted to “fastness” by adding the values inversely (1/x s) to account for the subjects unable to perform the test and scored as 0. The selected model is presented with the adjusted R^2^ statistic and the stability outcomes. A receiver operating characteristic (ROC)-analysis was conducted to evaluate the diagnostic accuracy of significant outcome parameters in the extended model for prediction of long-term participation.

In a second step, the identified model of sensorimotor outcomes was incorporated into a model with literature-based variables to test whether the found physical outcome measures remain significant. The additional variables were: age, sex, days at mechanical ventilation, sepsis, COVID-19 as the underlying illness, the presence of cerebral lesion, receipt of ECMO, Elixhauser Comorbidity Index [27], Barthel Index [30] and Montreal Cognitive Assessment (MoCA) [46] at V1, Hospital Anxiety and Depression Scale (HADS) [47] at V1. These patient demographics, illness and ICU-treatment characteristics have already been used to predict disability, frailty and morbidity in survivors of critical illness [1, 10, 21, 48]. The MoCA has previously shown an association with participation in persons with ICUAW [9] and was therefore added to account for the cognitive function at V1. Anxiety and depression, both assessed with the HADS, previously showed correlations with quality of life in critically ill patients [49]. The Barthel Index, a measure of activities of daily living, was considered due to its connection to participation or quality of life in other populations [18, 23].

A comparison between the extended model and the base model (including sensorimotor outcomes only), was performed using ANOVA.

Sample size for this study was based on the calculations reported in Bergmann et al. [26]. The recommendations of Heinze et al. [44] were followed, which suggest that statistical models with an events-per-variable ratio between 10 and 25 should involve post-estimation shrinkage methods and stability investigation.

Statistical analyses were conducted in RStudio (Version R 4.5.0), with the following packages: car, emmeans, ggplot2, lme4, lmerTest, nlme, performance, shrink, tidyr. The alpha level for statistical significance was set to 0.05.

### Data availability

The data that support the findings of this study are available from the corresponding author (JB), upon reasonable request. The data are not publicly available due to ethical restrictions.

## Results

**Fig. 2:**
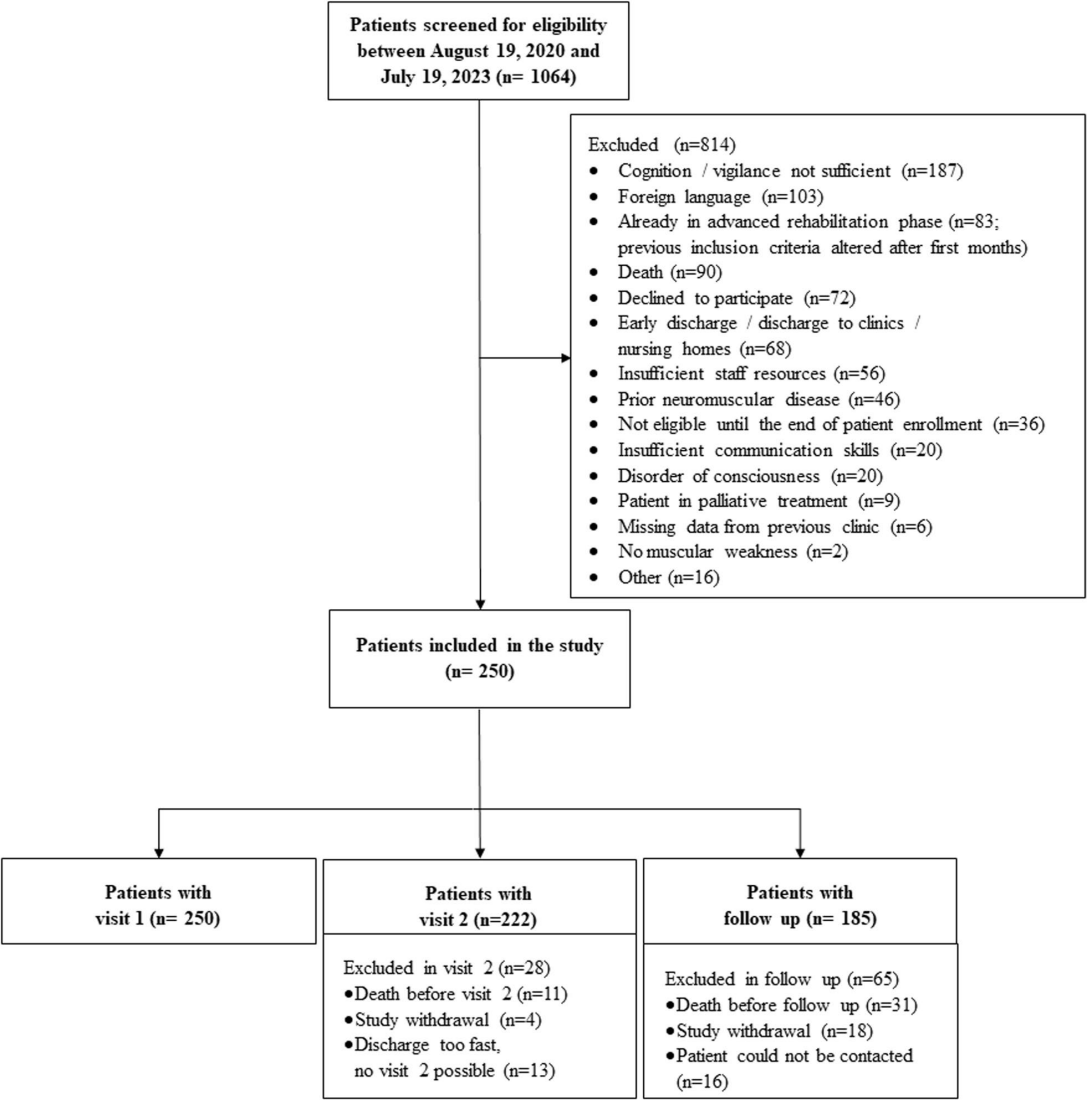
Flowchart

A total of 250 patients were included in this study. Details regarding exclusions for each study visit (V1, V2, FU) can be found in the flowchart (Fig. 2). For 12 participants, the FU was conducted as a telephone interview, resulting in a lack of data in performance-based sensorimotor outcomes.

All included patients had undergone prolonged mechanical ventilation and stayed a median of 55 days in the ICU (Table 1). The diagnoses for ICU-admission are presented in Table 1, with mainly COVID-19 (as the study was conducted in the peak of the pandemic in 2020–2021), beside cardiac and pulmonary diseases (together > 50%). About 99% of patients were able to walk without assistance from another person before admission to ICU, at least on even ground (90.0% FAC 5, 9.2% FAC 4), and preclinical frailty (Clinical Frailty Scale > 4) was observed in only 24 patients (9.6%).

**Table 1:** Characteristics of included patients

Characteristics	Total n = 250
Age, years	63 (54–73) min/max: 18/92
Sex, women	86 (34.4)
Length of hospitalization, days	141 (98–192); 156.4 ± 81.8
Length of ICU stay, days	55 (39–78); 64.7 ± 40.3
Length of mechanical ventilation, days	39 (27–58); 45.8 ± 31.1
Primary diagnosis	
COVID-19	67 (26.8%)
Cardiac disease	46 (18.4%)
Pulmonary disease	45 (18.0%)
Gastrointestinal/urological disease	25 (10.0%)
Bacterial infection	21 (8.4%)
Cerebral infarction/hemorrhage	20 (8.0%)
Polytrauma	8 (3.2%)
Oncological surgery	7 (2.8%)
Hypoxia	5 (2.0%)
Other	6 (2.4%)
Elixhauser comorbidity index	4.7 ± 7.0, min/max: − 7/28
ECMO	51 (20.4%)
Preclinical status	
Frailty (Clinical Frailty Scale)	3 (2–3)
Barthel index	100 (100–100), min/max: 60/100
Functional Ambulation Categories (FAC 0–5)	5 (5–5), min/max: 3/5
Preclinical somatosensory deficits	27 (10.8%)
Preclinical living conditions	
At home alone	52 (20.8%)
At home not alone (e.g., with family)	194 (77.6%)
Sheltered housing	2 (0.8%)
Preclinical occupation	
Employed	109 (43.6%)
Retired	124 (49.6%)
Student	3 (1.6%)
Unemployed	14 (5.6%)
Preclinical relationship	
Married/in a relationship	180 (72.0%)
Single	41 (16.4%)
Divorced	12 (4.8%)
Widowed	17 (6.8%)

Data are described as n (%), mean ± standard deviation or median (quartile 1-quartile 3)

*ECMO* extracorporeal membrane oxygenation; *ICU* intensive care unit

### Sensorimotor recovery

The development of each sensorimotor outcome parameter over 1.5 years is described in Table 2, with Table 3 presenting the mixed model estimates for change over time. Significant time effects were observed for all performance outcomes in the linear mixed model analysis, as well as in the global test for time effects (Table 3). Despite the significant improvements in the handgrip strength and strength in extremities, 65.4% of critical illness survivors had an MRC sum score below 48 at discharge from rehabilitation (compared to 86.7% at V1) and 25.7% still fulfilled this criterion for ICUAW 1.5 years after critical illness onset.

**Table 2:** Descriptive results of the sensorimotor outcomes throughout 1.5 years after disease onset

	Visit 1 (V1)	Visit 2 (V2)	Follow-up (FU)
Grip strength^a^	39.3 ± 17.6	51.6 ± 16.6	75.9 ± 22.1
MRC total (0–60)	40.0 (35.0–44.0)	45.0 (41.0–48.8)	54.0 (47.0–59.0)
MRC upper extremity (0–5)	3.5 (3.1–3.8)	3.8 (3.5–4.2)	4.7 (4.0–5.0)
MRC lower extremity (0–5)	3.2 (2.7–3.7)	3.7 (3.3–4.0)	4.5 (3.7–4.8)
FAC (0–5)	2.0 (0.0–3.0)	4.0 (3.0–5.0)	5.0 (3.8–5.0)
Mini-BESTest (0–28)	5.0 (0.0–15.0)	18.5 (10.0–23.0)	22.0 (16.0–25.0)
Functional Reach Test (cm)	0.0 (0.0–20.0)	23.0 (14.0–30.0)	26.0 (20.0–31.0)
Box-and-Block-Test^a^	70.6 (55.4–81.5)	84.0 (72.8–93.3)	91.4 (81.7–97.8)
5xSST^b^ (sec)	16.9 (14.1–22.5)	15.3 (12.1–19.8)	12.9 (10.6–17.1)
Number unable to perform the 5xSST	114 (46.9%)	19 (9.0%)	4 (2.4%)
Somatosensory deficits, all phenotypes	151 (60.9%)	117 (55.2%)	102 (59.3%)
Somatosensory deficits upper extremity	69 (27.8%)	40 (18.9%)	57 (33.1%)
Somatosensory deficits lower extremity	138 (55.6%)	105 (49.5%)	85 (49.4%)
Impaired light touch sense	83 (33.9%)	64 (32.0%)	61 (40.0%)
Impaired proprioception	26 (10.6%)	16 (7.7%)	13 (7.6%)
Impaired vibration sense	118 (47.8%)	81 (39.7%)	71 (41.3%)

Data are described as n (%), mean ± SD or median (quartile 1–quartile 3)

*MRC* Medical Research Council, *FAC* Functional Ambulation Categories, *5xSST* Five-Times-Sit-to-Stand-Test, *Mini-BESTest* Mini Balance Evaluation Systems Test

^a^ maximum of right or left, normed by reference (%). ^b,c^ Cases unable to perform this test were not included in median calculation

Due to missing values, sample sizes differ per assessment and time point as follows:

Grip strength V1 n = 247, V2 n = 217, FU n = 172;

MRC V1 n = 248, V2 n = 214, FU n = 171;

FAC V1 n = 250, V2 n = 222, FU n = 182;

Mini-BESTest V1 n = 246, V2 n = 212, FU n = 172;

Functional Reach Test V1 n = 243, V2 n = 210, FU n = 173;

5xSST V1 n = 129^c^, V2 n = 192^c^, FU n = 165^c^;

Box-and-Block-Test V1 n = 223, V2 n = 202, FU n = 171;

Sensation upper and lower extremity V1 n = 248, V2 n = 212, FU n = 172;

Sensation upper extremity V1 n = 248, V2 n = 212, FU n = 172;

Sensation lower extremity V1 n = 248, V2 n = 212, FU n = 172;

Light touch sense V1 n = 245, V2 n = 200, FU n = 165;

Proprioception V1 n = 246, V2 n = 209, FU n = 171;

Vibration sense V1 n = 247, V2 n = 204, FU n = 172

**Table 3:** Mixed model estimates of time effects for all sensorimotor outcomes

Outcome measure	Mixed model estimates [95% CI] (SE, p-value)			Global p-value
	V1—V2	V1—FU	V2—FU	
Grip strength^a^ (in %)	**11.89** [10.19, 13.6] (0.73, < 0.001)	**34.14** [30.82, 37.46] (1.41, < 0.001)	**22.25** [19.35, 25.15] (1.23, < 0.001)	< 0.001
MRC total	**5.46** [4.64, 6.29] (0.35, < 0.001)	**13.04** [11.9, 14.18] (0.49, < 0.001)	**7.58** [6.58, 8.58] (0.42, < 0.001)	< 0.001
MRC upper extremity	**0.39** [0.31, 0.47] (0.03, < 0.001)	**1.04** [0.94, 1.14] (0.04, < 0.001)	**0.65** [0.56, 0.74] (0.04, < 0.001)	< 0.001
MRC lower extremity	**0.52** [0.44, 0.61] (0.04, < 0.001)	**1.13** [1.02, 1.24] (0.05, < 0.001)	**0.61** [0.5, 0.71] (0.04, < 0.001)	< 0.001
FAC	**1.83** [1.6, 2.06] (0.1, < 0.001)	**2.27** [2.01, 2.54] (0.11, < 0.001)	**0.45** [0.29, 0.60] (0.07, < 0.001)	< 0.001
Mini-BESTest	**8.15** [7.04, 9.26] (0.47, < 0.001)	**10.65** [9.35, 11.95] (0.55, < 0.001)	**2.50** [1.6, 3.40] (0.38, < 0.001)	< 0.001
Functional reach test (cm)	**10.60** [8.87, 12.32] (0.73, < 0.001)	**13.84** [11.68, 16.01] (0.92, < 0.001)	**3.25** [1.56, 4.93] (0.72, < 0.001)	< 0.001
Box-and-block-test^a^ (in %)	**13.78** [10.65, 16.91] (1.33, < 0.001)	**19.99** [16.92, 23.06] (1.30, < 0.001)	**6.21** [4.25, 8.17] (0.83, < 0.001)	< 0.001
5xSST^b^ (sec)	**− 5.65** [− 7.31, − 4.00] (0.70, < 0.001)	**− 6.94** [− 8.98, − 4.91] (0.86, < 0.001)	− 1.29 [− 2.64, 0.06] (0.57, 0.066)	< 0.001
Sensory deficits, all phenotypes	0.68 [0.38, 1.23] (0.17, 0.28)	0.90 [0.48, 1.71] (0.25, 0.93)	1.33 [0.69, 2.55] (0.37, 0.57)	0.30
Somatosensory deficits upper extremity	**0.43** [0.21, 0.86] (0.13, 0.01)	1.48 [0.75, 2.92] (0.43, 0.37)	**3.48** [1.60, 7.56] (1.15, < 0.001)	< 0.001
Somatosensory deficits lower extremity	0.65 [0.65, 0.65] (0.00, < 0.001)	0.63 [0.63, 0.64] (0.00, < 0.001)	0.98 [0.98, 0.98] (0.00, < 0.001)	< 0.001
Impaired light touch sense	0.87 [0.47, 1.60] (0.23, 0.86)	1.26 [0.66, 2.39] (0.35, 0.69)	1.44 [0.73, 2.83] (0.42, 0.41)	0.44
Impaired proprioception	0.29 [0.07, 1.16] (0.17, 0.09)	0.36 [0.08, 1.57] (0.36, 0.23)	1.23 [0.26, 5.74] (0.81, 0.95)	0.08
Impaired vibration sense	0.58 [0.32, 1.08] (0.15, 0.10)	0.66 [0.34, 1.25] (0.18, 0.27)	1.12 [0.57, 2.19] (0.32, 0.92)	0.92

Data are presented as mean estimates or odds ratios (for sensory deficits) with [95% Confidence Interval] (Standard Error, p-value) for all sensorimotor outcomes and the p-value of the global time effect over all three time points (V1: Visit 1, V2: Visit 2, FU: follow-up)

*MRC* Medical Research Council, *FAC* Functional Ambulation Categories, *Mini-BESTest* Mini Balance Evaluation Systems Test, *5xSST* Five-Times-Sit-to-Stand-Test

^a^ Maximum of right or left, normed by reference (%). ^b^ Cases unable to perform this test were not included in mixed model calculation

Significant estimates or odds ratios are in bold

At the beginning of rehabilitation (V1), 34.8% of survivors of critical illness were unable to walk (FAC = 0), while 21.2% could walk independently at least over level surfaces (FAC ≥ 4). Throughout rehabilitation, FAC scores increased by almost two categories, reaching a median of FAC 5 [4, 5] at FU. Similarly, 54.7% initially were unable reaching forward in a standing position, and 36.2% could not perform any task on the Mini-BESTest at V1. However, improvements over time were significant for all balance outcomes as well. The number of patients unable to perform the 5xSST decreased as did time required to complete the five repetitions. Also, dexterity measured with the Box-and-Block-Test maximum reference values showed significant improvement. Regarding sensory testing, approximately 61% of the patients initially exhibited (V1) impaired sensation, which persisted at FU.

### Long-term participation

1.5 years after critical illness onset, good participation was achieved by 60.2% in this cohort. The median participation rate was 82% (64–95%). The distribution of the RNLI scores is illustrated in Supplementary Fig. 1.

Detailed results for the different statements of the RNLI are provided in Table 4. Notably, only 44% of individuals were most of their day occupied in a work activity that was necessary or important to them. Approximately 12% of the participants could not move around as desired within their community or living quarters and participation in social events was limited in over a third of individuals.

**Table 4:** Extent of agreement to reintegration to normal living Index statements

Statement	Full agreement	Partial agreement	No agreement
1) I move around my living quarters as I feel is necessary	137 (75.7%)	23 (12.7%)	21 (11.6%)
2) I move around my community as I feel is necessary	119 (65.7%)	39 (21.5%)	23 (12.7%)
3) I am able to take trips out of town as I feel are necessary	112 (61.9%)	43 (23.8%)	26 (14.4%)
4) I am comfortable with how my self-care needs (dressing, feeding, toileting, bathing) are met	131 (72.4%)	38 (21.0%)	12 (6.6%)
5) I spend most of my days occupied in a work activity that is necessary or important to me. (Work activity could be paid employment, housework, volunteer work, school, etc.)	79 (43.6%)	56 (30.9%)	46 (25.4%)
6) I am able to participate in recreational activities (hobbies, crafts, sports, reading, television, games, computers, etc.) as I want to	98 (54.1%)	65 (35.9%)	18 (9.9%)
7) I participate in social activities with family, friends, and/or business acquaintances as is necessary or desirable to me	117 (64.6%)	45 (24.9%)	19 (10.5%)
8) I assume a role in my family which meets my needs and those of other family members. (Family means people with whom you live and/or relatives with whom you don’t live but see on a regular basis	133 (73.5%)	35 (19.3%)	13 (7.2%)
9) In general, I am comfortable with my personal relationships	145 (80.1%)	22 (12.2%)	14 (7.7%)
10) In general, I am comfortable with myself when I am in the company of others	123 (68.0%)	42 (23.2%)	16 (8.8%)
11) I feel that I can deal with life events as they happen	120 (66.3%)	49 (27.1%)	12 (6.6%)

Adding to Statement 1)-3) Wheelchairs, other equipment or resources may be used; 4)-8) Adaptive equipment, supervision and/or assistance may be used

### Prediction of long-term participation with sensorimotor outcomes

The multiple linear regression model for the RNLI sum score at 1.5 years after critical illness onset included all sensorimotor outcome measures at V1 (see Supplementary Table 1 for correlation coefficients of sensorimotor outcome measures at FU and the RNLI at FU). After backward elimination, the Box-and-Block-Test, the 5xSST, the MRC sum score, and the Mini-BESTest remained in the selected model (Table 5). Only the Mini-BESTest total score reached significance, with the selected model yielding an adjusted R^2^ = 0.157 (p < 0.001). Notably, an increase of one point in the Mini-BESTest total score corresponded to a 0.85% increase in the RNLI total score. Results of the ROC-analysis are shown in Supplementary Fig. 2. The bootstrapping results supported the stability of the model, with similarity in estimates and bootstrap medians in the selected model and moderate selection frequencies for the four final outcome measures (57–68%).

**Table 5:** Multiple linear regression analysis for the physical outcomes only—full and selected model together with chosen bootstrap results

Variables (all at V1)	Full model			Bootstrap inclusion frequency (%)	Selected model			Bootstrap median	Bootstrap 95% CI	Relative bias^a^
	Beta coefficient	95% CI	p-value		Beta coefficient	95% CI	p-value			
Intercept	**41.99**	**17.55, 66.44**	** < 0.001**	100	**41.17**	**19.14, 63.21**	** < 0.001**	44.72	16.31, 76.57	7.75
Box-and-Block-Test^b^	0.17	− 0.02; 0.35	0.08	67.5	0.16	− 0.02, 0.35	0.08	0.18	0, 0.39	39.97
5xSST (1/sec)	− 138.42	− 306.81, 29.98	0.11	60.2	− 128.06	− 287.84, 31.71	0.12	− 127.76	− 284.34, 0	26.72
MRC sum score	0.56	− 0.15, 1.27	0.12	59.2	0.55	− 0.11, 1.21	0.10	0.55	0, 1.27	43.51
Mini-BESTest score	0.65	− 0.36, 1.66	0.21	57.2	**0.85**	**0.2, 1.51**	**0.01**	0.59	0, 1.52	46.84
Functional Reach Test	0.27	− 0.32, 0.86	0.37	40.8				0	0, 0.88	107.82
Sensation deficit (yes)	− 0.72	− 8.41, 6.97	0.85	25.2				0	− 9.25, 7.38	376.47
Grip strength^b^	− 0.02	− 0.27, 0.23	0.85	21.1				0	− 0.28, 0.27	8.96
FAC (≥ 4, independent)	− 2.77	− 14.74, 9.20	0.65	17.4				0	− 12.21, 8.19	133.66
R^2^	0.184				0.178					
Adjusted R^2^	0.141				0.157					
Residual Std. Error	21.07 (df = 150)				20.87 (df = 154)					
F-Statistic	4.234 (df = 8; 150); p < 0.001				8.352 (df = 4; 154); p < 0.001					

*95% CI* 95% Confidence Interval, *df* Degrees of Freedom

^a^ Relative bias conditional on selection

^b^ Maximum of right or left, normed by reference (%)

*V1* Visit 1 at start of rehabilitation, *MRC* Medical Research Council, *Mini-BESTest* Mini Balance Evaluation Systems Test, *FAC* Functional Independence Categories, *5xSST* Five-Times-Sit-to-Stand-Test

The bootstrap median is zero in case a variable was chosen in < 50% of the resamples. Significant values are in bold

In the second step, the selected model including sensorimotor measures was added to the model including demographic and clinical factors. Employing the variable selection algorithm, the variables depression, duration of mechanical ventilation, MoCA, Mini-BESTest, Elixhauser Comorbidity Index, sex, and cerebral lesion were included in the selected model. The detailed estimates, R^2^ statistic and the stability outcomes are presented in Table 6. The inclusion frequencies were the highest for depression, the duration of mechanical ventilation and the MoCA (> 90%), followed by the Mini-BESTest with 88%. Each additional point in the depression score was associated with a 1.8% decrease in participation, each additional day of mechanical ventilation with a 0.2% decrease, while each additional point on the MoCA was associated with a 1.1% increase in the RNLI total score. Being female and suffering from a cerebral lesion was associated with a decrease in participation by about 7%.

**Table 6:** Multiple linear regression analysis for the extended model—the full and selected model together with chosen bootstrap results

	Full model			Bootstrap inclusion frequency (%)	Selected model			Bootstrap median	Bootstrap 95% CI	Relative bias^a^
Variables	Beta coefficient	95% CI	p-value		Beta coefficient	95% CI	p-value			
Intercept	42.52	2, 83.05	0.04	100	65.69	43.14, 88.24	< 0.001	46.44	7.76, 87.62	9.87
Depression	− 1.99	− 3.02, − 0.97	< 0.001	99.9	− 1.78	− 2.56, − 1	< 0.001	− 1.94	− 2.95, − 1.07	− 2.08
Duration of mechanical ventilation (days)	− 0.20	− 0.3, − 0.1	< 0.001	97.6	− 0.22	− 0.31, − 0.13	< 0.001	− 0.2	− 0.32, − 0.07	3.25
MoCA	1.32	0.32, 2.31	0.01	91.7	1.06	0.21, 1.91	0.02	1.27	0, 2.32	3.91
Mini-BESTest	0.73	0.09, 1.37	0.03	88	0.46	0.12, 0.8	0.01	0.66	0, 1.22	-0.28
Elixhauser Comorbidity Index	− 0.49	− 0.98, 0	0.05	73.8	− 0.45	− 0.88, − 0.02	0.04	− 0.47	− 0.96, 0	18.39
Sex (male)	5.88	− 0.55, 12.3	0.07	71.7	7.06	0.96, 13.17	0.02	6.06	0, 12.23	29.14
5xSST (1/sec)	− 99.13	− 249.95, 51.69	0.2	55				− 99.81	− 219.04, 0	44.91
Age (years)	0.17	− 0.11, 0.44	0.24	47.2				0	0, 0.44	58.73
Cerebral lesion (yes)	− 5.1	− 15.79, 5.59	0.35	37.3	− 7.82	− 16.49, 0.86	0.08	0	− 14.96, 0	95.6
MRC sum score	0.26	− 0.34, 0.87	0.39	36				0	0, 0.94	120.69
ECMO (yes)	0.82	− 7.11, 8.74	0.84	31.1				0	− 8.4, 10.75	227.05
Box-and-Block-Test^b^	− 0.03	− 0.23, 0.16	0.73	24.7				0	− 0.21, 0.22	25.55
Anxiety	0.23	− 0.72, 1.19	0.63	22.2				0	− 0.65, 1.18	183.87
Sepsis (yes)	0.41	− 6.18, 6.99	0.90	20.6				0	− 5.63, 7.05	250.27
Barthel Index	− 0.02	− 0.26, 0.21	0.85	18.1				0	− 0.24, 0.19	271.73
COVID-19 (yes)	− 1.06	− 8.65, 6.53	0.78	17.1				0	− 8.20, 6.10	117.4
R^2^	0.401				0.385					
Adjusted R^2^	0.334				0.357					
Residual Std. Error	18.55 (df = 142)				18.23 (df = 151)					
F-Statistic	5.947 (df = 16; 142); p < 0.001				13.510 (df = 7; 151); p < 0.001					

95% Confidence Interval; *df* Degrees of Freedom. Variables at start of rehabilitation (Visit 1) or baseline

*ECMO* Extracorporeal membrane oxygenation, *MoCA* Montreal Cognitive Assessment, *Mini-BESTest* Mini Balance Evaluation Systems Test total score

^a^Relative bias conditional on selection

^b^Maximum of right or left, normed by reference (%)

The bootstrap median is zero in case a variable was chosen in < 50% of the resamples. Significant values are in bold

Overall, the model largely met the core assumptions of linear regression, although with some limitations. Diagnostic plots indicated deviations from linearity, slight heteroscedasticity, and minor deviations from the normality of residuals. However, there was no indication of problematic multicollinearity or highly influential observations. Details can be found in Supplementary Figs. 3 and 4. Post-estimation shrinkage factors for both selected models are displayed in Supplementary Table 2.

A comparison of two regression models using ANOVA revealed that the extended model significantly improved the fit compared to the simpler model with solely sensorimotor outcomes, as indicated by a substantial reduction in residual sum of squares from 67,083 to 50,198. The F-test showed a significant improvement in model fit (F(3, 151) = 16.931, p < 0.001), suggesting that the inclusion of additional predictors in the extended model contributed meaningfully to explaining variance in participation.

## Discussion

This study investigated the recovery of sensorimotor function in survivors of critical illness during inpatient rehabilitation and in the long-term, as well as their level of participation 1.5 years after disease onset, and the predictive value of early sensorimotor functions for long-term participation. Consistent with our hypothesis, we observed significant improvements in sensation, muscle strength, balance, and dexterity over time. However, persistent deficits were evident at both rehabilitation discharge and at 1.5-year follow-up, indicating that full recovery of sensorimotor function remains incomplete in a substantial proportion of survivors. At 1.5 years post-onset, the median participation rate was 82%, with 60.2% of participants reaching a “good” participation level, defined as a score > 75% among individuals with ICUAW. A multivariable model incorporating the Mini-BESTest, Box-and-Block Test, 5 × Sit-to-Stand Test (5xSST), and MRC sum score significantly predicted participation, albeit explaining only 15.7% of the variance. These findings suggest that, although sensorimotor function contributes to participation, other factors, including psychological, cognitive, demographic, and potentially social determinants, also play a significant role.

### Sensorimotor recovery

The mixed model analysis revealed significant and clinically meaningful improvements across nearly all sensorimotor outcomes over time. Notably, recovery extended beyond the inpatient rehabilitation phase, although the rate of improvement declined thereafter. Gains in muscle strength, postural balance, and upper limb dexterity indicate a favorable trajectory for both neuromuscular control and coordination during and following structured rehabilitation. Improvements in functional mobility also support enhanced lower extremity performance and walking capacity over the long term.

By rehabilitation discharge, balance performance approached levels reported in community-dwelling individuals with neurological conditions, suggesting that substantial recovery is achievable in a clinical setting [19, 20]. Despite these encouraging gains, a considerable proportion of participants continued to exhibit clinically relevant impairments 1.5 years after critical illness. This finding underscores the importance of extending rehabilitation efforts and monitoring beyond the inpatient phase. Further research is needed to investigate how these long-term impairments in survivors of critical illness can be effectively targeted. Outpatient care structures may play a crucial role in enhancing sensorimotor functions after the rehabilitation period.

Sensation testing revealed that over one-third of the participants exhibited deficits in either light touch, vibration, or both, while impairments in proprioception were less common. Overall, approximately 60% of participants demonstrated sensation deficits, most commonly in the lower extremities, at both ICU discharge and long-term follow-up. About 11% of participants reported pre-existing sensory impairments prior to hospital admission, suggesting a substantial proportion of the sensory deficits may be attributed to the critical illness or ICU stay. Mold et al. [50] documented age-related increases in bilateral sensory deficits, most notably absence of the ankle reflex and diminished touch and vibration sensitivity. The prevalence of at least one bilateral sensory deficit in elderly increased from 26% in individuals aged 65–74 years to 54% in those aged 85 and older. Previous studies have shown that sensory loss in critically ill patients is frequently associated with pain and tends to persist long-term, with little improvement over time [16, 51]. Taken together, these findings highlight a critical and under-addressed component of post-ICU recovery. There is an urgent need for targeted therapeutic strategies that explicitly address sensory deficits, potentially through neurosensory retraining or novel rehabilitation technologies.

### Long-term participation

This study offers novel insights into long-term social participation in survivors of critical illness, an outcome domain that remains underexplored. At 1.5 years post-illness, 25% of participants reported not being regularly engaged in meaningful or necessary daily tasks. Other common limitations involved mobility around living quarters, community and taking trips. Given that the majority of survivors regained independent walking ability (FAC ≥ 4), this finding suggests that ambulation alone is not sufficient to ensure meaningful social participation.

To date, participation in individuals with ICU-acquired weakness (ICUAW) has been primarily reported at 6- and 12-month follow-ups, with RNLI scores of 65.5% and 68.2% [9]. In our study, median RNLI scores were higher at 18 months, potentially indicating continued recovery over time. This trend requires validation in future studies but may reflect late-stage gains that are not captured in shorter follow-ups. Participation levels in our cohort were comparable to those reported in neurological populations such as stroke and multiple sclerosis, highlighting similar long-term challenges across conditions affecting sensorimotor and cognitive function [19, 20].

Our classification of “good participation” was based on the ≥ 75% RNLI cut-off as used by Thomas and Mehrholz et al. [9], though this threshold has not been formally validated. The only validated cut-off to date (87.5%) has been used to distinguish chronic stroke patients from healthy controls [52]. Consequently, caution is warranted in interpreting these classifications, and there is a clear need for disease-specific, validated thresholds in survivors of critical illness.

Participation is closely linked to health-related quality of life [12]. A recent study reported a moderate correlation of the RNLI and the EQoL-5D-3L in survivors of sepsis [13]. While generic health-related quality of life measures such as the EQ-5D-3L primarily assess basic functional outcomes, they may overlook the psychosocial and existential dimensions of returning to meaningful living after hospital discharge which is an essential concern for survivors of critical illness [13, 53]. Notably, returning to a “normal” life does not imply resuming a pre-illness state but rather adapting to new or altered living conditions, which often involves renegotiating personal, social, and occupational roles [12, 13]. Several studies report that health-related quality of life in ICU survivors remains significantly lower than in the general population, even years after discharge, despite partial recovery over time [54–56]. For instance, a recent study of mechanically ventilated patients with COVID-19 found lower quality of life over a 2-year period particularly among individuals with multiple coexisting impairments [57]. Factors such as older age, delirium, and prolonged mechanical ventilation were associated with poorer health-related quality of life, whereas male sex, living with family, and ECMO treatment predicted better outcomes.

Predicting participation in the present study was restricted to an explorative approach, since its individual and complex nature bears many uncertainties. We found that early physical performance was a limited predictor of long-term participation. A model incorporating the Mini-BESTest, Box-and-Block Test, 5 × Sit-to-Stand Test, and MRC sum score accounted for only 15.7% of participation variance. When expanded to include additional variables—duration of mechanical ventilation, depression, cognitive function, comorbidities, sex, and cerebral lesion—the model explained 35.7% of the variability. This highlights the multifactorial and individualized nature of participation outcomes.

The Mini-BESTest emerged as a relevant predictor, warranting further investigation into which subcomponents, such as anticipatory postural adjustments, reactive balance, or sensory orientation, are most strongly linked to participation. Previous research in stroke patients has shown that tandem stance specifically affected participation most strongly [17]. It is possible that improved proprioceptive and reactive balance confer a sense of physical security, which is critical for social and community engagement. Fear of falling, actual falls, and pain have all been associated with reduced participation in older adults and individuals with physical disabilities [58–60]. While visual impairments and pain were not assessed in the present analysis, they are known to be prevalent in survivors of critical illness and may negatively affect balance and, by extension, participation [16]. These dimensions represent important targets for future research and clinical assessment.

Psychological and cognitive factors also played a key role in participation outcomes. We observed a strong association between depressive symptoms and reduced participation. Estrup et al. [61] reported that approximately 8% of ICU survivors experienced persistent depressive symptoms one year post-discharge, with little change from the 3-month mark. These findings are consistent with other reports documenting lasting reductions in quality of life and significant associations between depression, executive dysfunction, and poor mental health outcomes following critical illness [61–63]. In our cohort, general cognitive performance was also strongly associated with participation. This aligns with previous studies in ICUAW, stroke, and multiple sclerosis populations, where cognition has been linked to balance, instrumental activities of daily living, and community reintegration [9, 19, 20, 64].

Mechanical ventilation duration was another key determinant, reinforcing previous findings that longer ventilation times are associated with worse functional outcomes and higher mortality [2, 4, 10]. Notably, we also found that female sex was independently associated with reduced participation, with a 7% lower rate compared to males. Sex differences in participation have not been previously reported in survivors of critical illness, and no such effect was observed in stroke cohorts [18, 20]. This finding highlights a potentially understudied factor that merits further investigation [59].

In sum, social participation following critical illness is influenced by a complex interplay of physical, psychological, cognitive, and demographic factors. While early rehabilitation assessments provide some predictive value, they are not sufficient to fully capture participation outcomes. Clinicians may enhance long-term participation by addressing modifiable barriers, including balance deficits, cognitive dysfunction, depressive symptoms, and persistent sensorimotor impairments, through tailored interventions across inpatient and community settings.

## Limitations

Several limitations should be acknowledged in this study. Firstly, the sensorimotor recovery and prediction of participation observed in our cohort may not be representative of all survivors of critical illness. Our cohort had a prolonged duration of ICU treatment and mechanical ventilation compared to other cohorts [10, 21], potentially influencing the outcomes and generalizability. Additionally, being a monocentric study with a high percentage of patients with COVID-19 as the underlying illness, generalizability may be limited. Multicenter studies involving a broader range of patients, including those who were somnolent or unable to communicate at the beginning of rehabilitation, are warranted to validate our findings.

Another limitation is the insufficient evaluation of psychometric properties for most outcome measures. Though commonly used in critical care research, these measures lack thorough assessment of reliability and validity. Future studies are needed to ensure their robustness and accuracy.

The regression results should be interpreted cautiously, as variable selection can introduce uncertainty and affect validity. To address this, we followed Heinze et al. [44] using backward elimination with AIC as the stopping criterion, post-estimation shrinkage, and model stability checks. However, external validation was not performed, which could have offered additional insights into the model’s robustness and generalizability. Moreover, sensation impairment was included in the regression model despite showing only a low correlation with the RNLI at FU, as this is an explorative study. This may have influenced the model.

Although dropout rate was comparably small, taking this vulnerable population with high mortality rates into account [8–10, 21], missing data across various tests and visits posed challenges during data analysis. Missing data were treated missing at random in the mixed model analysis, what might not be fully adequate.

## Conclusions

This study provides important insights into long-term sensorimotor recovery and social participation in survivors of critical illness. While substantial improvements in sensation, strength, balance, and dexterity were observed over 1.5 years post-illness onset, persistent deficits remained common and only 60.2% of participants achieved good participation levels, underscoring the ongoing challenges many survivors face in reintegrating into daily life.

Early rehabilitation assessments of sensorimotor function showed limited predictive power for long-term participation. A multivariable model including the Mini-BESTest and other sensorimotor tests explained 15.7% of the variance in participation. When expanded to include psychological, cognitive, demographic, and clinical factors (e.g., depression, cognitive function, duration of mechanical ventilation, comorbidities, sex, and cerebral lesions), the model accounted for 35.7% of the variance. These findings highlight the multifactorial nature of social participation and suggest that additional determinants, including psychosocial and environmental factors, warrant further investigation.

A comprehensive understanding of sensorimotor recovery and its limited yet meaningful association with participation enables clinicians to set realistic expectations and develop individualized, multidisciplinary interventions. Future efforts should aim to enhance long-term outcomes by addressing both physical and non-physical barriers to participation in survivors of critical illness.

## Supplementary Information

Supplementary material 1: Figure 1 Distribution of the Reintegration to Normal Living Index (RNLI) total score. Table 1 Correlation coefficients for the sensorimotor outcome measures at follow-up and the Reintegration to Normal Living Index at follow-up. Figure 2 Receiver Operating Characteristic (ROC) curve illustrating the predictive performance of the MiniBESTest for distinguishing between good and poor long-term participation. The area under the curve (AUC) is 0.6727 (95% CI 0.595–0.751), indicating fair discriminatory ability. The optimal threshold of 9.5, determined using the Youden Index, yields a sensitivity of 0.546 and a specificity of 0.792. Figure 3 Model Performance of the selected model with physical outcomes Linear model with Box-and-Block-Test, Five-Times-Sit-to-Stand-Test, Mini Balance Evaluation Systems Test (MiniBEST) and the muscle strength measured by the Medical Research Council (MRC) sum score at Visit 1 (V1) describing the Reintegration of Normal Living Index in % (RNLI_p). Figure 4 Model Performance of the extended selected model linear Model with depression, duration of mechanical ventilation, sex, cerebral ischemia, Elixhauser comorbidity index, Mini Balance Evaluation Systems Test (MiniBEST), and Montreal Cognitive Assessment (MoCA) describing the Reintegration of Normal Living Index in % (RNLI_p). Table 2 Parameterwise shrinkage factors and shrinkage-adjusted estimates.

## Data Availability

The datasets used and/or analyzed during the current study are available from the corresponding author on reasonable request.

## Abbreviations

AIC: Akaike information criterion
ANOVA: Analysis of variance
CINAMOPS: Critical Illness Polyneuropathy and Myopathy: Outcome, Predictors and Longitudinal Trajectories
ECMO: Extracorporeal membrane oxygenation
FAC: Functional Ambulation Categories
FU: Follow-up
HADS: Hospital Anxiety and Depression Scale
ICU: Intensive care unit
ICUAW: Intensive care unit-acquired weakness
Mini-BESTest: Mini Balance Evaluation Systems Test
MoCA: Montreal Cognitive Assessment
MRC: Medical Research Council
PICS: Post-intensive care syndrome
RNLI: Normal Living Index
V1: Visit 1
V2: Visit 2
5xSST: Five-Times-Sit-to-Stand-Test
