# Supplementary material for: Long-term recovery of sensorimotor functions and prediction of participation in survivors of critical illness: a prospective cohort study
Source: J Intensive Care. 2025 Sep 8;13:49. doi: 10.1186/s40560-025-00808-9 (PMC12418612; doi:10.1186/s40560-025-00808-9)
Supplement: Supplementary file 1 — Supplementary material 1: Figure 1 Distribution of the Reintegration to Normal Living Index (RNLI) total score. Table 1 Correlation coefficients for the sensorimotor outcome measures at follow-up and the Reintegration to Normal Living Index at follow-up. Figure 2 Receiver Operating Characteristic (ROC) curve illustrating the predictive performance of the MiniBESTest for distinguishing between good and poor long-term participation. The area under the curve (AUC) is 0.6727 (95% CI 0.595–0.751), indicating fair discriminatory ability. The optimal threshold of 9.5, determined using the Youden Index, yields a sensitivity of 0.546 and a specificity of 0.792. Figure 3 Model Performance of the selected model with physical outcomes Linear model with Box-and-Block-Test, Five-Times-Sit-to-Stand-Test, Mini Balance Evaluation Systems Test (MiniBEST) and the muscle strength measured by the Medical Research Council (MRC) sum score at Visit 1 (V1) describing the Reintegration of Normal Living Index in % (RNLI_p). Figure 4 Model Performance of the extended selected model linear Model with depression, duration of mechanical ventilation, sex, cerebral ischemia, Elixhauser comorbidity index, Mini Balance Evaluation Systems Test (MiniBEST), and Montreal Cognitive Assessment (MoCA) describing the Reintegration of Normal Living Index in % (RNLI_p). Table 2 Parameterwise shrinkage factors and shrinkage-adjusted estimates. [file 40560_2025_808_MOESM1_ESM.docx]

Long-term Recovery of Sensorimotor Functions and Prediction of Participation in Survivors of Critical Illness Survivors – A Prospective Cohort Study

Johanna WEGHORN ^1,2^, Melanie FINSTERHÖLZL ^2^, Franziska WIPPENBECK^2^, Klaus JAHN^2,3^, Marion EGGER ^2*^, Jeannine BERGMANN^2,3*^

*These authors share last authorship.

^1^ Physiotherapy, School of Health Professions, Bern University of Applied Sciences, Bern, Switzerland; ^2^ Research Group, Department of Neurology, Schoen Clinic Bad Aibling, Bad Aibling, Germany; ^3^ German Center for Vertigo and Balance Disorders, Ludwig-Maximilians-Universität (LMU), University Hospital Grosshadern, Munich, Germany

Corresponding author: Jeannine Bergmann, Research Group, Department of Neurology, Schoen Clinic Bad Aibling, Kolbermoorer Strasse 72, 83043 Bad Aibling, Germany. E-mail: jbergmann@schoen-klinik.de


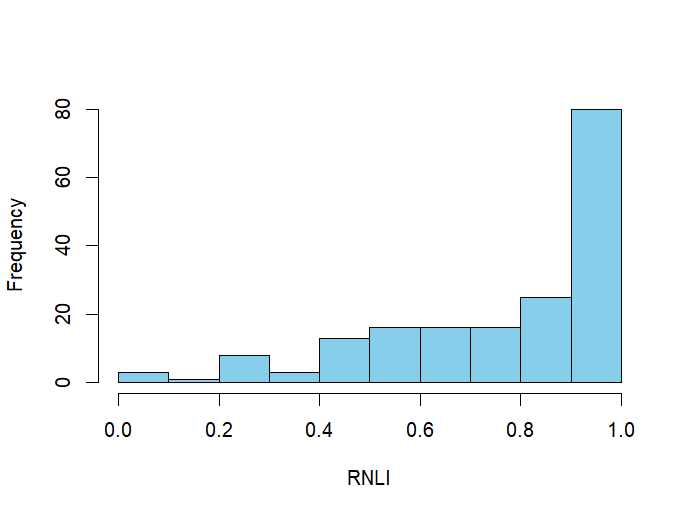


**Supplementary Figure 1** Distribution of the Reintegration to Normal Living Index (RLNI)

**Supplementary Table 1** Correlation coefficients for the sensorimotor outcome measures at follow-up and the Reintegration to Normal Living Index at follow-up.

| **Assessment** | **Correlation coefficient (Spearman)** |
| --- | --- |
| MiniBESTest | 0.674 |
| Medical Research Council sum score | 0.604 |
| Functional Ambulation Categories | 0.577 |
| Five Times Sit to Stand Test | -0.533 |
| Functional Reach | 0.532 |
| Grip strength in % of reference value | 0.502 |
| Box-and-Block-Test | 0.428 |
| Sensation deficits, all phenotypes | -0.222 |

**
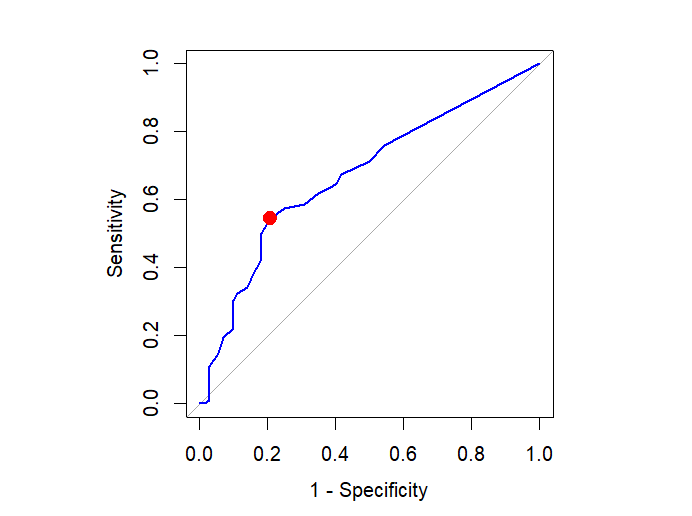
**

**Supplementary Figure 2** Receiver Operating Characteristic (ROC) curve illustrating the predictive performance of the MiniBESTest for distinguishing between good and poor long-term participation. The area under the curve (AUC) is 0.6727 (95% CI: 0.595–0.751), indicating fair discriminatory ability. The optimal threshold of 9.5, determined using the Youden Index, yields a sensitivity of 0.546 and a specificity of 0.792.


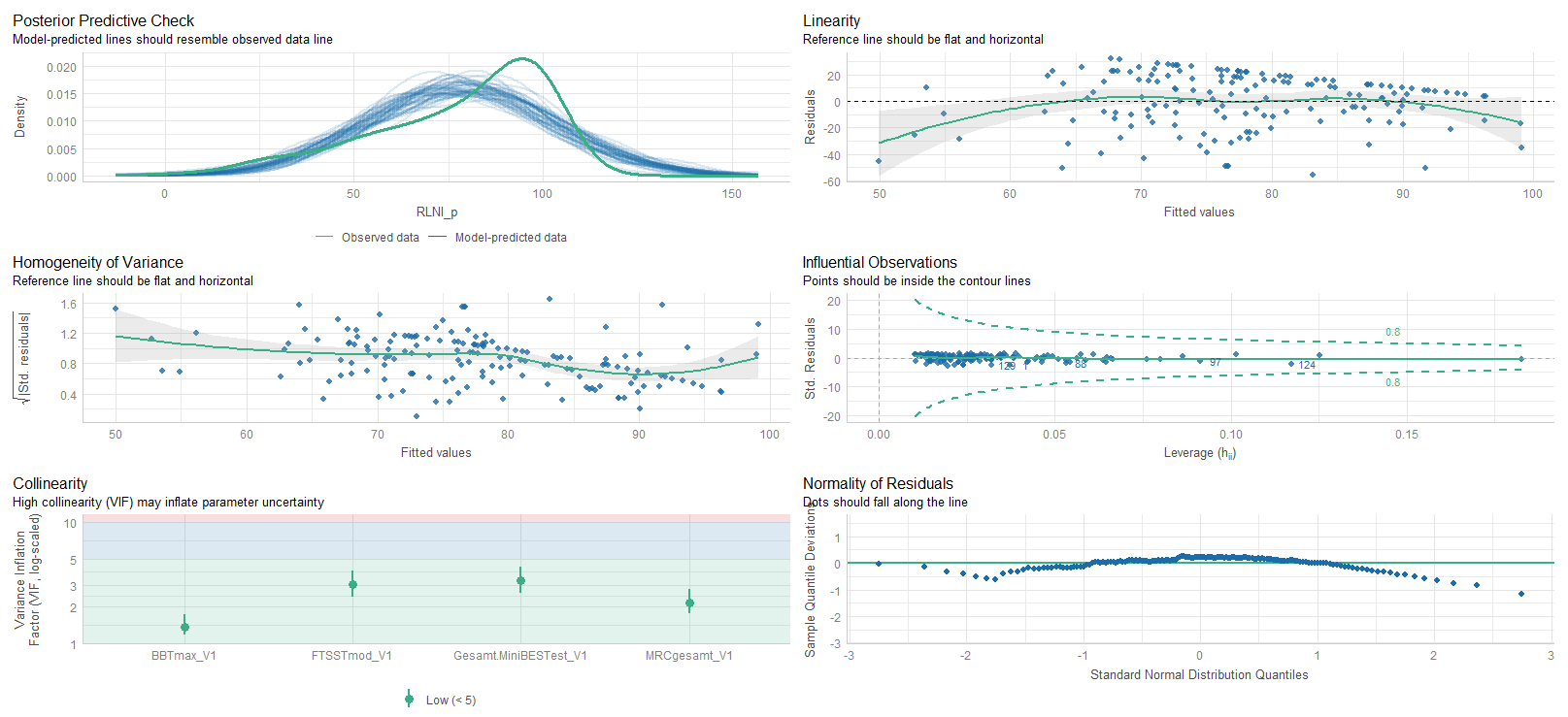


**Supplementary Figure 3** Model Performance of the selected model with physical outcomes
Linear model with Box-and-Block-Test, Five Times Sit to Stand Test, Mini Balance Evaluation Systems Test (MiniBEST) and the muscle strength measured by the Medical Research Council (MRC) sum score at Visit 1 (V1) describing the Reintegration of Normal Living Index in % (RLNI_p).


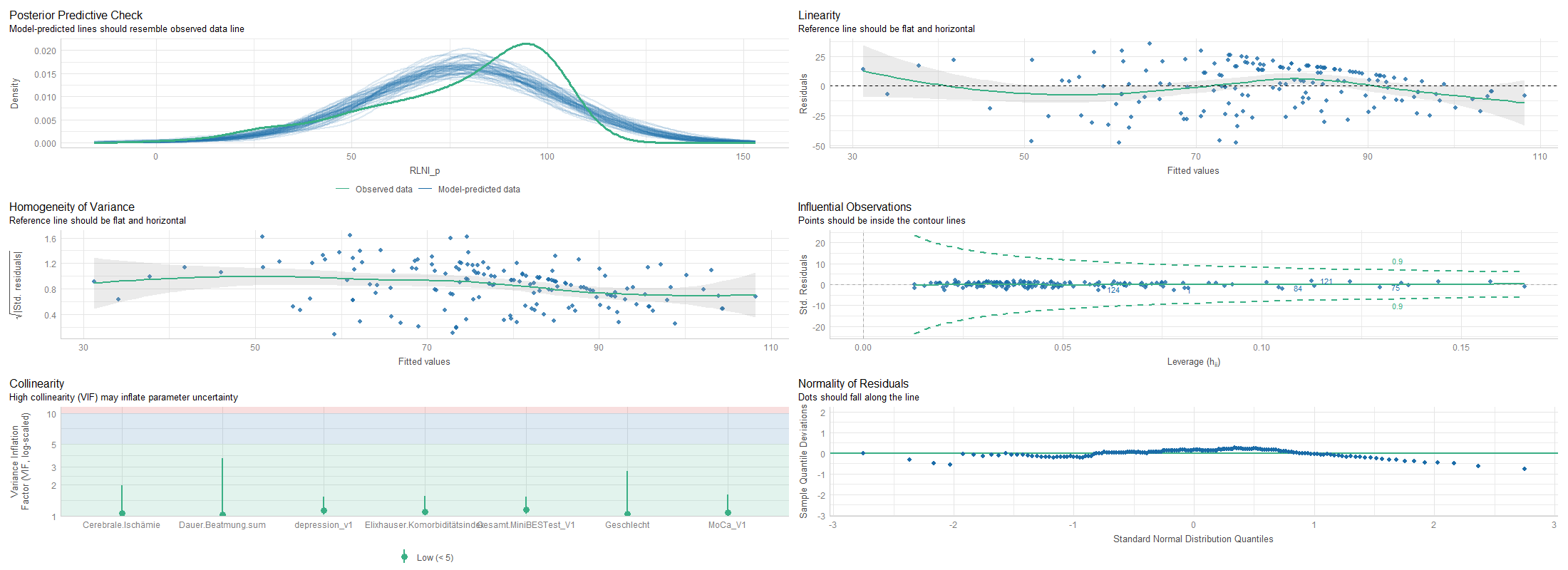


**Supplementary Figure 4** Model Performance of the extended selected model
Linear Model with depression, duration of mechanical ventilation, sex, cerebral ischemia, Elixhauser comorbidity index, Mini Balance Evaluation Systems Test (MiniBEST), and Montreal Cognitive Assessment (MoCA) describing the Reintegration of Normal Living Index in % (RLNI_p).

| **Supplementary Table 2** Parameterwise shrinkage factors and shrinkage-adjusted estimates | | | | | |
| --- | --- | --- | --- | --- | --- |
| **Model with sensorimotor variables** | | | **Model with extended variables** | | |
| **Variable** | **Parameterwise shrinkage factor** | **Shrinkage-adjusted estimate** | **Variable** | **Parameterwise shrinkage factor** | **Shrinkage-adjusted estimate** |
| Box-and-Block-Test | 0.647 | 0.106 | Depression | 0.956 | -1.702 |
| 5xSST (1/sec) | 0.484 | -61.92 | Duration of  mechanical ventilation (days) | 0.924 | -0.202 |
| MRC sum score | 0.713 | 0.392 | MoCA | 0.970 | 1.025 |
| Mini-BESTest score | 0.896 | 0.763 | Mini-BESTest | 1.018 | 0.472 |
|  |  |  | Elixhauser Comorbidity Index | 0.612 | -0.275 |
|  |  |  | Sex (male) | 0.797 | 5.628 |
|  |  |  | Cerebral lesion (yes) | 0.590 | -4.616 |
